# Regulating the 20S Proteasome Ubiquitin-Independent Degradation Pathway

**DOI:** 10.3390/biom4030862

**Published:** 2014-09-23

**Authors:** Gili Ben-Nissan, Michal Sharon

**Affiliations:** Department of Biological Chemistry, Weizmann Institute of Science, Rehovot 7610001, Israel; E-Mail: Gili.Ben-Nissan@weizmann.ac.il

**Keywords:** protein degradation, 20S proteasome, intrinsically disordered proteins, oxidatively damaged proteins, regulatory mechanisms

## Abstract

For many years, the ubiquitin-26S proteasome degradation pathway was considered the primary route for proteasomal degradation. However, it is now becoming clear that proteins can also be targeted for degradation by the core 20S proteasome itself. Degradation by the 20S proteasome does not require ubiquitin tagging or the presence of the 19S regulatory particle; rather, it relies on the inherent structural disorder of the protein being degraded. Thus, proteins that contain unstructured regions due to oxidation, mutation, or aging, as well as naturally, intrinsically unfolded proteins, are susceptible to 20S degradation. Unlike the extensive knowledge acquired over the years concerning degradation by the 26S proteasome, relatively little is known about the means by which 20S-mediated proteolysis is controlled. Here, we describe our current understanding of the regulatory mechanisms that coordinate 20S proteasome-mediated degradation, and highlight the gaps in knowledge that remain to be bridged.

## 1. Introduction

Cells must continuously adapt their proteome composition [1]. The constant flux of the proteome is reflected in the fact that most proteins have a half-life several fold shorter than cell generation time, even for rapidly dividing cells [2]. Protein level adjustment in response to various conditions such as oxidative stress, cellular differentiation and fluctuating nutrient supply is critical for cell viability. The control of protein levels relies not only on the regulation of protein production and folding, but also on protein degradation. Failure or malfunction of this process may lead to various illnesses, including cancer and neurodegenerative diseases.

**Figure 1:**
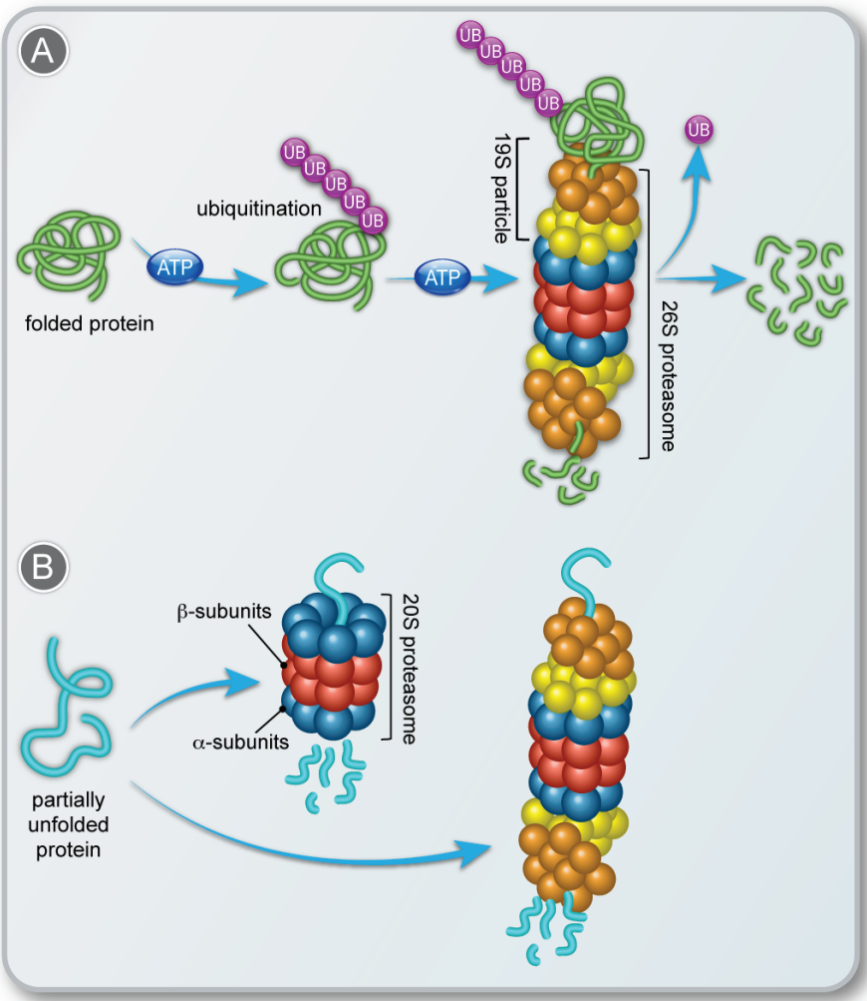
A simplified representation of the (**A**) ubiquitin-dependent 26S proteasome; and (**B**) ubiquitin- and ATP-independent 26S or 20S proteasome, protein degradation pathways.

The ubiquitin-dependent 26S proteasomal degradation process is the major system that mediates the degradation of damaged and short-lived regulatory proteins (for reviews, see [3,4,5]) (Figure 1A). This active, ATP-dependent pathway involves a cascade of three different types of enzymes that tag substrate proteins with an ubiquitin chain, for their recognition and proteolysis by the 26S proteasome. The structure of the 26S proteasome consists of two entities: a 20S core particle, and a 19S regulatory particle (Figure 1A). The 20S complex carries out the proteolytic activities. It is composed of 28 subunits encoded by 14 different genes, 7α and 7β subunits, which form a highly conserved cylindrical structure of four rings, each containing seven subunits. The two outer rings contain α-type subunits whose function is to maintain a “gate” through which proteins enter the catalytic site [6]. The β-subunits form the two inner rings. Three of the β-subunits, β1, β2 and β5, are catalytically active, and responsible for the caspase-like (or peptidylglutamyl-peptide), trypsin-like and chymotrypsin-like hydrolyzing functions, respectively. Overall, this architecture creates a compartment whose proteolytic active sites are restricted to its interior, so that only proteins entering this chamber are degraded. The addition of a 19S regulator to either one or both ends of the 20S proteasome creates the 26S proteasome. The 19S particle coordinates the degradation process by recognizing the polyubiquitinated substrates, unfolding, deubiquitinating and translocating them into the interior of the 20S complex, where they are degraded to oligopeptides.

Recently, it was realized that proteins are also subject to proteasomal degradation in an ubiquitin-independent manner [7,8,9]. Both the 26S and the 20S proteasomes were shown to be capable of degrading proteins without the substrate selectivity achieved by ubiquitin tagging (Figure 1B). The primary requirement for degradation by this route is the presence of an unstructured region. In fact, the two alternative proteasomal degradation mechanisms, ubiquitin-dependent and ubiquitin-independent, are not mutually exclusive, and different populations of the same protein can be sent to degradation via either pathway [10]. Here, we will focus on the ubiquitin-independent degradation process that is mediated only by the 20S proteasome complex, without the assistance provided by the 19S particle.

Substrates of the 20S proteasome are made up of proteins that have become partially or completely unfolded due to aging, mutations or oxidation [8,11]. Native proteins containing large unstructured segments (>30 amino acids in length), referred to as intrinsically disordered regions (IDRs), or proteins with entirely disordered sequences (intrinsically disordered proteins, IDPs) [12], are also susceptible to degradation by the 20S proteasome (see summary in Table 1). This latter group of substrates is dominated by key regulatory and signaling proteins with essential roles in cell cycle progression, cellular growth control, and oncogenesis (as reflected in Table 1) [13]. Thus, their levels must be tightly controlled, as alterations in their abundance may lead to the development of various diseases [11].

**Table 1:** A list of proteins with varying degrees of disorder that serve as 20S proteasome substrates.

Protein	Function	Associated Disease	% Disorder ^a^	Reference
p21 ^cip1^	Cell cycle regulator	Cancer	57 (64)	[14,15]
p27 ^kip1^	Cell cycle regulator	Cancer	98 (104)	[16]
p33	Tumor suppressor	Cancer	77 (115)	[17]
p53	Tumor suppressor	Cancer	62 (128)	[10]
p63	Tumor suppressor	Cancer	51 (73)	[18,19]
p73	Tumor suppressor	Cancer	33 (57)	[10]
Rb	Tumor suppressor	Cancer	35 (73)	[20]
BIM_EL_	Pro-apoptotic protein	Cancer	38 (62)	[21]
MCL-1	Anti-apaptotic protein	Cancer	30 (105)	[22]
c-Fos	Transcription factor	Cancer	33 (88)	[23]
IκBα	NF-κB inhibitor	Cancer	43 (77)	[24]
ODC	Biosynthesis of polyamines	Cancer	15 (32)	[25]
HIF-1α	Transcription factor	Cancer	55 (108)	[26]
C/EBPα	Transcription factor	Myeloproliferative disease	40 (70)	[27]
PGC-1α	Transcription co-activator	Neurological disorders	96 (178)	[28]
tau	Neuronal protein, stabilizes microtubules	Alzheimer’s	80 (165)	[29,30]
α-synuclein	Presynaptic neuronal protein	Parkinson’s	31 (44)	[31]

^a^ Values represent the percentage of disordered residues as predicted by the FoldIndex program [32]. Numbers in parenthesis indicate the amino-acid length of the longest unfolded stretch.

Considering that 44% of human protein-coding genes contain IDRs [12], it is reasonable to assume that in the future, the number of proteins recognized as substrates of the ubiquitin-independent pathway will continue to increase. That being said, it should be noted that establishing whether a protein is degraded in a 20S proteasome ubiquitin-independent manner is not a trivial task. *In vitro* assays showing that the non-ubiquitinated protein is degraded by the 20S proteasome cannot stand on their own, and should be complemented by cellular assays, as detailed in recent reviews [7,33]. In practice, however, even successful demonstration of *in vivo* ubiquitin-independent degradation cannot unambiguously distinguish between 20S- or 26S-mediated degradation. Results indicating that the protein of interest: (i) is not ubiquitinated upon destabilization or inhibition of the proteasome; (ii) is not stabilized following mutations in potential ubiquitin acceptor sites, or due to the excess of non-polymerizable ubiquitin; and (iii) is not stabilized when inhibiting the ubiquitination pathway, which imply that either 20S and/or 26S proteasome ubiquitin-independent degradation has taken place. In order to define which of the proteasomes is involved in the process, approaches indicating whether the 19S particle is involved in the process should be used.

Several recent findings emphasize the significance of the ubiquitin independent 20S proteasome degradation route. First, the majority of proteasomes in mammalian cells were found to be uncapped 20S proteasomes, whereas only about 20%–30% of the total number of mammalian proteasomes were identified as 26S proteasomes [34,35,36,37]. Secondly, more than 20% of the cellular proteins in mammalian cell extracts were shown to undergo 20S proteasomal cleavage [38], suggesting the extensive involvement of this process in cell viability. Moreover, under oxidizing conditions, the 20S proteasome was identified as the major degradation machinery (reviewed in [8,11,39]). The 20S proteasome was shown to be more resistant to oxidative stress and to maintain its activity under conditions in which protein damage occurs, while the 26S proteasome and the ubiquitination pathway are much more vulnerable to such oxidative stress conditions. In summary, these findings suggest that 20S-mediated proteolysis is not limited to rare cases, but rather represents a complementary degradation route that is critical for removing damaged, unfolded proteins, and for maintaining normal levels of proteins containing IDRs.

Over the past three decades, extensive research focusing on the ubiquitin-dependent 26S proteasome degradation pathway has uncovered hundreds of factors that regulate this process—including a series proteins that deliver the ubiquitinated substrates to the 26S proteasome, enzymes that antagonize degradation by trimming off the ubiquitin chain, and proteasome-associated ubiquitin ligases that enhance substrate turnover [3,4,5,6]. In contrast, relatively little is known about the mechanisms that coordinate protein degradation by the 20S proteasome. Here, we will discuss the emerging view of how the proteolytic capacity of the 20S proteasome is specifically controlled. In doing so, we will describe eight distinct mechanisms that enable either enhancement or inhibition of 20S proteolytic capacity, including: the disassembly of the 26S proteasome, gene regulation, subunit composition diversity, and associations with regulatory and substrate-targeting molecules, as well as post-translational modifications (PTMs) and divergence in cellular localization. We also examine how temporal regulation is critical for forming a tightly coordinated and dynamic system for combating oxidative stress.

## 2. Molecular Mechanisms that Regulate the Function of the 20S Proteasome

### 2.1. Disassembly of the 26S Proteasome

Cellular levels of the 20S proteasome can be increased through disassembly of the 26S proteasome into its 20S and 19S components. Several studies have shown that this, indeed, occurs following oxidative stress, at the time when there is an urgent need to enhance the degradation capacity of this complex so as to cope with increasing numbers of oxidatively damaged proteins [40,41,42,43]. Various proteins appear to be involved in the decomposition process. In the mammalian system, the chaperone Hsp70 was found to be important for the stabilization of the 19S regulator after its dissociation from the 20S proteasome, and for the reassembly of functional 26S proteasomes, once the oxidative stress is eliminated [41]. In yeast, H_2_O_2_-induced disassembly of 26S proteasomes is dependent on the proteasome-associated protein, Ecm29 [40]. Low levels of Hsp90 were also shown to cause almost complete disassembly of the yeast 26S proteasome, and an increase in the abundance of free 20S proteasomes [44]. Inevitably, the release of the 20S proteasome from the holo-26S complex is accompanied by a reduction in ATP-stimulated peptidase activity; however, this did not appear to influence the degree of resistance to oxidative conditions [42,45,46].

Recently, the levels of the 26S and 20S proteasomes were also shown to be dependent on the cellular metabolic state [43,47]. For example, a low cellular ratio of the cofactors NADH/NAD^+^ destabilizes the 26S proteasome, giving rise to free 20S proteasome particles [47]. Similarly, ATP deficiency associated with acute and long-term mitochondrial impairment leads to a reduction in 26S proteasome levels, in concert with an increase in 20S proteasomes [42]. Overall, the disassembly of the 26S proteasome is a straightforward mechanism for increasing 20S levels. However, further research is needed, in order to specifically indicate whether the increase in the 20S levels is accompanied by a concomitant rise in the catalytic capacity of the 20S proteasome. If true, such a process could ensure efficient degradation of damaged proteins and proteins with IDRs, without the energy expenditure associated with ubiquitin-dependent 26S-mediated degradation.

### 2.2. Alternative Proteasome Activators

More than 50% of cellular 20S proteasomes are found in a free form, and about 30% are associated with the 19S regulatory particle. The remaining 20S proteasome pool is associated with other proteasome activators (PAs)—the cytosolic PA28αβ (or 11S), as well as the nuclear PA28γ and PA200 regulators [35,48]—that enhance the proteolytic capacity of the complex in an ATP- and ubiquitin-independent manner. Both PA28αβ and PA28γ form hetero- or homo-heptameric rings, respectively, which interact with the α-rings of the 20S proteasome. This association activates the proteasome by triggering structural rearrangements that lead to the opening of the sealed gate structure of the complex [49,50,51]. PA200 is a monomer, but similar to the PA28 complexes, it docks onto the 20S proteasome orifice, and results in gate opening [52].

Activation of the 20S proteasome by PA28αβ was initially associated with the immune response since, like the immunoproteasome, PA28αβ is more abundant in immune tissues and is upregulated by interferon, suggesting that it has a role in antigen presentation [53]. The nuclear proteasome activator, PA28γ, was shown to activate the 20S both *in vitro* and *in vivo*, as demonstrated in the degradation of the nuclear factor p21 [14,54] and the oncogenic steroid receptor coactivator-3 (SRC-3) [55]. This activation, though, appears to be selective, since PA28γ was unable to enhance the degradation of another unstructured cell cycle regulator, p27 [14].

More recently, it was realized that during adaptation to oxidative stress, the 20S immunoproteasome and the PA complexes were up-regulated, whereas the level of the 19S proteasome remained unchanged [55]. PA28 regulators were also tied to the clearance of oxidized proteins under stress conditions. PA28αβ and PA28γ increased the capacity of the 20S proteasome to degrade oxidized proteins, and PA28αβ could also induce the degradation of oxidized proteins by means of the immunoproteasome [43]. Similarly, in the process of embryonic stem cell differentiation, a significant increase in the levels and peptidase activity of the immunoproteasome and PA28αβ was seen, accompanied by reduced levels of carbonylated proteins, whereas silencing of PA28αβ caused accumulation of damaged proteins [56]. The role of PA200 in the regulation of 20S degradation is less straightforward, as on the one hand, PA200 was shown to enhance the 20S degradation of acetylated core histones [57], and the yeast ortholog of PA200, Blm10, enhanced degradation of the partially unstructured Dmn1 protein, a major regulator of mitochondrial morphology [58]. On the other hand, PA200 actually inhibited the proteolytic capacity of both the 20S proteasome and the immunoproteasome towards oxidized proteins [43]. Thus, it is possible that the PA200 particle and the PA28 regulators possess distinct functionalities.

The alternative proteasome activators exhibit yet another level of regulation, by forming highly complex interactions, as they appear to bind to the 20S proteasome, with or without the addition of a 19S regulator [36,43,59,60,61]. These associations generate multiple types of hybrid proteasomes that stimulate the degradation of non-ubiquitinated substrates [34,36,43,56,59,60,62,63] and possibly also ubiquitinated substrates. Future studies are likely to clarify how the structural diversity of the proteasome population enables compartments, cells or tissues to rapidly respond to various physiological conditions.

### 2.3. Gene Regulation

An increase in the proteolytic activity of the 20S proteasome can also be achieved by up-regulation of 20S subunit expression. Several studies involving *Drosophila melanogaster*, *Caenorhabditis elegans*, and mammalian cells have indicated that the increase in 20S proteasome expression is mediated by Nrf2, the master regulator of antioxidant transcriptional responses, following oxidative stress [64]. The synthesis of 20S proteasome subunits was shown to facilitate H_2_O_2_-induced adaptation to oxidative stress. A microarray-based survey, however, identified the Nrf2 pathway as enhancing the expression of both 19S and 20S proteasome subunits [65,66]. Further analysis indicated that sothiocyanate sulforaphane (SFN), an Nrf2 activator, specifically up-regulates the levels of the 20S proteasome’s catalytic β-subunits, leading to enhanced proteolytic activity in murine neuroblastoma cells [67]. Hence, while it is broadly accepted that stabilization of Nrf2 induces the expression and activation of 20S proteasomes, it remains to be determined whether increased expression of 19S subunits also plays a functional role in combating the oxidative stimulus.

Recently, another set of studies have demonstrated that induction of proteasome gene expression is also mediated by the related transcription factor, Nrf1 [68,69]. This transcription factor was shown to up-regulate the expression of both 20S and 19S genes in an Nrf2-independent manner, in response to proteotoxic stress caused by proteasome inhibition. In the context of proteasomal gene activation, Nrf1 and Nrf2 appear to have only partially redundant roles, as their functions vary among cell types, tissues and cellular stresses [68,69,70,71]. Interestingly, Nrf1 is present in the cell in several alternative splicing forms, which have different cellular functions; furthermore, it was shown that the post-translational processing of Nrf1 is mediated by the 26S proteasome complex [72]. Thus, inhibition of the proteasome may result in a response favoring accumulation of the full-length variant, which in turn results in the induction of proteasome gene expression [72,73].

### 2.4. Variations in Subunit Composition

Another means to regulate the 20S proteasome function is through varying the building blocks of which it is composed. Indeed, a number of studies demonstrate that substantial diversity exists in the subunit composition of the 20S proteasome population. The most common variants are the immunoproteasome catalytic subunits: LMP2 (β_1i_), MECL-1 (β_2i_), and LMP7 (β_5i_), which can replace the conventional catalytic β-subunits (β_1_, β_2_ and β_5_) in different combinations (reviewed in [34,74]). Immunoproteasome subunits are constitutively expressed at low levels in various cell types, and at higher degrees in the immune and lymphatic tissues. In addition, they are up-regulated and incorporated into newly assembled 20S proteasomes in various tissues, in response to specific stimuli such as cytokine treatment, viral, fungal or bacterial infection, or injury, as well as ischemia reperfusion and muscle atrophy due to aging or diabetes ([62,75,76], and references within).

The major function of the immunoproteasome was traditionally thought to encompass the processing of specific peptides which would serve as antigenic peptides for MHC class I complex antigen presentation [75]. In recent years, however, the immunoproteasome has also emerged as an important player in the removal of damaged proteins in response to stress conditions [77,78,79]. This view is supported by the fact that the immunoproteasome was shown to be up-regulated during conditions of oxidative stress [78], and that depletion of the immunoproteasome from cells results in higher susceptibility and lower adaptation to such a state [77,78]. Moreover, when associated with 20S regulators such as the PA28αβ complex, immunoproteasomes display higher specific activity, as well as a greater capacity to degrade oxidized proteins [43].

In addition to the immunoproteasome, several other, tissue-specific 20S subunits have been identified, including the thymoproteasome and spermatoproteasome subunits [76]. The 20S proteasomes in the thymus predominantly contain a specific β_t5_ subunit, and are thought to be primarily associated with antigenic peptide generation for positive selection of T lymphocytes [80], while the mammalian testis-specific proteasomes contain an alternative α_4s_ subunit, implicated together with the nuclear PA200, in the degradation of acetylated histones during spermatogenesis and DNA repair [57,81,82]. Interestingly, both the thymo- and spermatoproteasome subunits can be found together with the immunoproteasome β_1i_ and β_2i_ subunits within the 20S proteasome (reviewed in [62]). These observations imply that various combinations of the 20S particle can be generated, however, the functional contributions of these divergent sets of proteasomes, and their unique, tissue-specific roles, if any, require further study.

### 2.5. Interacting Regulatory Proteins

Like other protein complexes, the function of the 20S proteasome can be modulated by interactions with regulatory proteins. To date, only a small number of studies demonstrating protein association-mediated enhancement or inactivation of the 20S proteasome’s proteolytic activity are available. These include the proteasome’s associations of NAD(P)H: quinone oxidoreductase 1 and 2 (NQO1 and NQO2), Hsp90, and Poly [ADP-ribose] polymerase 1 (PARP-1) (detailed below); however, our own research indicates that additional proteins that specifically regulate 20S proteasome function exist [83].

NQO1 is a stress-inducible flavoprotein that, in addition to its role as an antioxidant enzyme, was shown to physically bind the 20S proteasome [84] and protect IDPs from degradation [10]. These include p33, p53, p63, p73α, c-Fos, C/EBPα and PGC-1α [10,17,18,19,23,27,28,85]. It seems that the regulatory role of NQO1 is conserved throughout evolution, as the yeast ortholog, Lot6, was also reported to bind the 20S proteasome and control degradation in a redox-dependent manner [86]. Apart from its association with the proteasome, NQO1 has been shown in several instances to bind the IDP substrates in an NADH-dependent manner [10,27,28], or even to compete with the 20S proteasome for substrate interaction [27]. However, the exact molecular mechanism underlying NQO1’s ability to inhibit substrate degradation remains uncertain.

Cellular levels of NQO1 greatly increase in response to various stimuli, including oxidative stress, UV light, and ionizing radiation [87]. This up-regulation of NQO1 seems to regulate the levels of proteins containing IDRs, as was shown for p53 and p73 [88] and, more recently, for the myeloid differentiation factor, C/EBPα, following exposure of mice to γ-radiation [27]. Thus, while a correlation exists between NQO1 and the levels of partially folded proteins, it is still not known how up-regulation of NQO1 influences the 20S proteasome-mediated degradation of oxidatively damaged proteins. Overloading the 20S proteasome with oxidatively damaged proteins may clog the proteasome and inhibit degradation. Proteasomal dysfunction may then lead to decreased degradation of misfolded proteins, resulting in accumulation of oxidized proteins, and subsequent protein aggregation. It is possible that by preventing protein degradation, NQO1 regulates the flux of proteins which are targeted to the 20S proteasome, so as to maintain proper proteasomal function. Thus, NQO1 may be part of a system that interrupts this cascade of events, which otherwise could lead to various ailments, especially neurodegenerative diseases.

Recently, we have shown that the function of NQO1 is also coordinated by the levels of its redox cofactor, FAD, forming a link between cellular metabolic status and protein stability [84]. When NQO1 is bound to FAD, it adopts a compact folded structure; however, in the absence of FAD binding, the apo form occupies a partially unfolded conformation and becomes a substrate of the 20S proteasome, like any other intrinsically unstructured protein. Thus, a double negative feedback loop between NQO1 and the 20S proteasome exists, whereby on the one hand, NQO1 can prevent the proteolytic activity of the proteasome, while on the other hand, the proteasome regulates NQO1 levels by degrading its apo state.

Interestingly, another oxidoreductase, NRH:quinone oxidoreductase 2 (NQO2 ) was also shown to rescue proteins with IDRs from 20S proteasome degradation [89,90]. Like NQO1, NQO2 is also induced by stress, and its cellular levels are increased by exposure to carcinogens and ionizing radiation. Accordingly, its up-regulation leads to stabilization of regulatory proteins containing IDRs such as p53 and C/EBPα, in dependence on its electron donor, dihydronicotinamide riboside (NRH). NQO2 is thought to inhibit degradation by interaction with substrates, and its protective role appears to be independent of NQO1 [89,90]. Thus, it is tempting to speculate that oxidoreductases and possibly other structurally-related proteins take part in the concerted regulation of the stability of proteins comprising IDRs.

An additional protein that controls the catalytic capacity of the 20S proteasome is Hsp90. This molecular chaperone is one of the most abundant proteins in eukaryotic cells, comprising 1%–2% of total cellular proteins, even under basal conditions [91]. Hsp90, besides its role in stabilizing damaged proteins and preventing their aggregation [92], directly binds the 20S proteasome and influences its activity [93,94,95]. This interaction has been implicated in promoting the degradation of oxidatively damaged proteins [93], and may play a role in protecting the 20S proteasome from oxidation inactivation [96,97]. How Hsp90 influences 20S proteasome activity, and whether other chaperones aid in the recognition and targeting of substrates to the 20S proteasome, is still unclear.

During oxidative stress, a vast increase in proteasomal activity is detected in the nucleus, as early as 15 min after exposure. This enhanced proteolytic function is correlated with the presence and activity of PARP-1, and mediated by poly(ADP-ribosyl)ation [98,99]. In response to DNA damage, especially single-strand DNA breaks, PARP-1 modifies nuclear proteins by poly ADP-ribosylation, probably as a means of DNA damage signaling, to induce its repair [100]. Polymers of ADP-ribose, as well as auto-modified PARP-1, were shown by *in vitro* studies to interact non-covalently with the 20S proteasome, leading to an increase in proteasomal peptidase activity [101]. Likewise, inhibition of PARP-1 activity leads to a decrease in nuclear proteolytic activity [98]. Still to be determined is whether, in addition to oxidative stress, other DNA damaging conditions may also lead to PARP-1-mediated 20S proteasome activation. Nevertheless, these studies implicate PARP-1 as an important player in the proteasome-dependent removal of oxidation-damaged proteins in the nucleus. This conclusion is supported by the fact that decreasing PARP-1 activity during aging correlates with the decline of nuclear proteasome activity, and accumulation of oxidized nuclear proteins [102].

### 2.6. Substrate-Targeting Proteins

Several accessory proteins that are either transiently or constitutively expressed, can recognize and target specific substrates for degradation by the 20S proteasome. This has been shown for the cell cycle regulator p21: due to its crucial cellular task, several mechanisms have evolved to tightly regulate its abundance, both by ubiquitin-dependent and -independent routes [15,103]. In the latter instance, the so-called accessory protein MDM2 was shown to promote the 20S proteasome-mediated degradation of p21, by enhancing the association between p21 and the proteasome α_7_ subunit [104,105,106,107]. Paradoxically, during this process, MDM2, an ubiquitin E3 ligase, actually promotes ubiquitin-independent degradation. Recently, it was discovered that the 14-3-3τ protein also facilitates this process [108]. The 14-3-3t protein promotes complex formation with MDM2, p21, and the 20S proteasome α_7_ subunit, triggering the degradation of the free p21 pool during the G_1_ phase. Considering that enhanced degradation of p21 leads to unchecked cell cycle progression and proliferation, it is not surprising that overexpression of MDM2 and 14-3-3τ was found to be correlated with cancerous processes [108,109].

This strategy of targeting proteins to the 20S proteasome for their degradation has also been harnessed by viruses, which employ the host cell’s protein degradation machinery for their own use [7]. For example, IκBα, a regulator of NFκB-mediated transcription [110], is targeted for degradation by the viral protein Tax, which is encoded by the human T-cell leukemia virus (HTLV-1) [111,112]. Tax was found to promote the interaction between IκBα and two subunits of the 20S proteasome, α_3_ and β_4_, probably leading to the degradation of IκBα, and the transformation and survival of cells infected with HTLV-1. Similarly, Tax was shown to associate with the tumor suppressor retinoblastoma (Rb) protein and target it for proteasomal degradation; however, further investigation is necessary, to determine whether the 26S or the 20S proteasome is involved in this process [113].

### 2.7. Post-Translational Modifications that Influence 20S Proteasome Activity

An additional approach to modifying the function of protein complexes is through post-translational modifications (PTMs). Various proteasome subunits have been reported to undergo phosphorylation, as well as acetylation, myristoylation, ubiquitination, modification with *O*-linked *N*-acetyl-glucosamine (*O*-GlcNAc), *S*-glutathionylation, and oxidation [3,114]. However, here we will only describe PTMs that can specifically and reversibly modulate 20S proteasome function.

In this regard, the tyrosine kinases c-Abl and Arg were reported to interact with the proteasome α_4_ subunit and phosphorylate it, leading to inhibition of proteasomal peptidase activity [115]. In contrast, association of the α_3_ and α_4_ subunits with the polo-like kinase (Plk), resulting in their phosphorylation, seems to increase the 20S proteasome proteolytic activity [116]. Moreover, reduction in the phosphorylation levels of α_3_ and α_7_ was shown to promote the disassembly of the 26S proteasome into 19S and 20S particles [117,118]. In a similar fashion, stress induction by interferon-γ, which triggers a decrease in the phosphorylation of both 20S and 19S subunits, leads to the disassembly of 26S proteasomes, and enhanced 20S proteasomal peptidase activity [118].

*S*-glutathiolation of cysteine residues within α subunits was found to affect 20S proteasome activity in yeast [119,120,121]. It appears that oxidation of Cys residues to sulfenic acid species, followed by glutathiolation, induces gate opening in the 20S proteasome, and consequently enhances degradation of damaged and partially unstructured proteins. This post-translational modification can be reversed by oxidoreductases. In yeast, the major glutaredoxin, Grx2, as well as the cytoplasmic thioredoxins 1 and 2, deglutathionylate the 20S proteasome [121]. Overall, *S*-glutathiolation is emerging as an important mechanism for coping with stressful conditions, enabling transient and reversible adaptation of the proteolytic machinery.

### 2.8. Cellular Localization

One major regulatory mechanism within cells is compartmentalization, thereby regulating the sub-cellular localization of the various cellular components. The 20S proteasome is considered to be ubiquitously distributed within the cell; however, it specifically accumulates within different sub-cellular domains. The majority of 20S proteasomes are found in the cytoplasm; a smaller sub-fraction is also associated with the cytoskeleton [122,123] and the microsomal fractions [34,124,125]. A lesser amount of 20S proteasome is present in the nucleus, where it localizes within the nucleoplasm, but not within the nucleoli. In addition, 20S proteasomes are also associated with different nuclear domains, such as perinuclear centrosome-associated structures [126], matrix-associated nuclear bodies [125], and interchromatin nuclear speckles [127].

Notably, several studies have demonstrated the redistribution of proteasomes in response to different cues. In solid tumors, for example, stress conditions such as glucose starvation or hypoxia induce the recruitment of proteasomes to the nucleus [128], and in various types of cancerous cells, proteasomes were shown to accumulate on chromatin, together with PA200, in response to ionizing radiation [129]. Similarly, in MEF cells, proteasomes were shown to be recruited to mitochondria during mitochondrial depolarization [130]. Although such studies cannot always accurately differentiate between the 20S and the 26S proteasomes, it is only reasonable to assume that the cell possesses various regulatory mechanisms capable of relocalizing either particle towards specific locations, when needed.

Recent studies have also identified active 20S proteasomes in various extracellular compartments, such as serum, cerebrospinal fluid, and extracellular bronchoalveolar lavage fluids. Moreover, the levels of these so-called “circulating” or extracellular proteasomes are significantly elevated under stress conditions such as injury and various diseases, as well as in different types of cancer [131,132,133,134,135,136]. Circulating proteasomes were shown to be predominantly in the form of 20S particles but it remains to be established whether these extracellular proteasomes also act in association with regulatory complexes. The function of 20S proteasomes in the extracellular fluids is not clear, but they may be involved in the clearance of proteins outside of the cellular context, particularly in instances of stress, in the process of extracellular antigen processing, or even in a novel, “moonlighting” role, outside of the cellular boundaries. Taken together, the 20S proteasome appears to be impressively omnipresent both within and outside of the cellular context, and regulation of its localization is part of the cell’s ongoing efforts to maintain homeostasis, as well as to cope with stress.

## 3. Dynamic Regulation Following Oxidative Stress

Clearly, the aforementioned regulatory mechanisms are intertwined, as exemplified by their coordinated adaptation to oxidative stress [8,11]. Prior to the onset of stress, the 26S proteasome functions as the major degradation machinery. Following oxidative stress, the proteolytic capacity of the 20S proteasome is enhanced to rapidly remove oxidatively damaged proteins, thus preventing the pathogenicity caused by protein aggregates. Thus, within half an hour of oxidative stress, the activity of pre-existing 20S proteasomes is significantly elevated. In the nucleus, PARP-1 is involved in this process [98,99,101]; other activators may exist in the cytoplasm. A more persistent activation challenge (3–5 h) leads to a reduction in 26S proteasome activity, accumulation of ubiquitinated substrates and oxidized products, and an increase in 20S proteasome activity. The latter is due to the disassembly of the 26S proteasome, which, in turn, liberates active 20S particles [40,41]. After combating the oxidative stress, reassembly of the 26S proteasome takes place, and degradation of ubiquitinated substrates resumes [40].

Throughout sustained exposure to oxidative stress (at least 12 h), *de novo* synthesis of both constitutive and immuno-20S proteasomes occurs [78,79]. The newly generated 20S complexes can function on their own, or associate with the PAs and/or 19S regulatory complexes to form various proteasome assemblies [78]. Overall, temporal regulation enables a coordinated and accelerated response to take place, depending on the length and severity of the stress.

The fate of proteins comprising IDRs during this dynamic enhancement of 20S proteasome activity is still not known. It could be that their degradation is inevitable or, alternatively, that mechanisms have evolved to maintain their cellular abundance during the enhanced 20S catalytic process. The latter was recently demonstrated for α-synuclein, in which oxidation of two methionine residues under mild oxidation conditions prevented its degradation [137]. The 20S-mediated degradation of α-synuclein was restored, following enzymatic reversal of the oxidation. This observation might suggest that while oxidative conditions induce the transition from a natively folded to a partially unstructured state in some proteins, making them prone to 20S proteasome degradation, oxidation in IDPs may lead to structural stabilization, thus reducing their proteolytic susceptibility.

## 4. Conclusions and Perspectives

**Figure 2:**
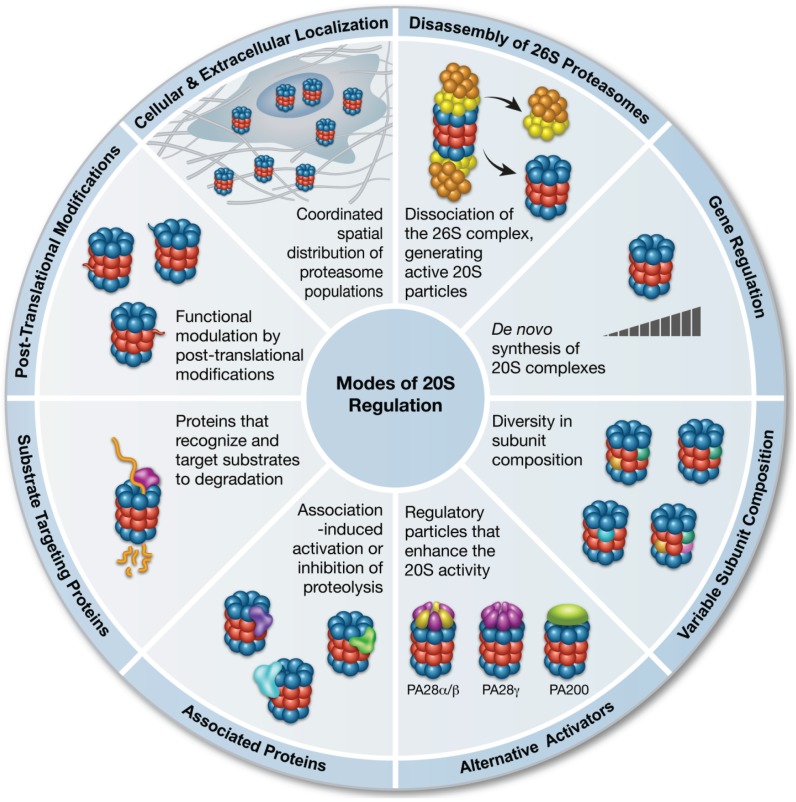
Specific mechanisms that regulate the function of the 20S proteasome.

As research into the 20S proteasome activity and ubiquitin-independent degradation continues, new insights are helping to unravel the multiple mechanisms that coordinate this pathway (Figure 2). These intricate regulatory modes, and the key role played by the 20S proteasome system during oxidative stress, strongly suggest that 20S-mediated proteolysis is not an incidental occurrence, but rather an independent and highly complementary degradation route. It is reasonable to speculate that this degradation pathway is especially crucial at times when rapid elimination of proteins is required for maintaining cellular viability. Overcoming the enzymatic cascade required for ubiquitination not only enhances the rate of degradation, but also spares the energy costs associated with it. Thus, under basal conditions, proteolysis by the 20S proteasome may not constitute a dominant route for protein turnover; however, under stress conditions, the 20S proteasome degradation capacity is significantly enhanced, as well as assisted by its ATP and ubiquitin-independent regulators, PA28 αβ, PA28γ and PA200. Overall, there are probably multiple occasions in which switching off programmed degradation of native ubiquitin-tagged proteins by the 26S proteasome, thereby favoring 20S-mediated degradation, are essential for maintaining protein homeostasis.

Considering the narrow entrance of the 20S proteasome, and the lack of the 19S regulatory particle that causes proteins to unfold, substrates of this degradation route are primarily limited to partially unfolded proteins that can access its restricted opening. The two main groups of substrates that fit this category and were shown to be susceptible to 20S-mediated degradation are: the natively existing proteins with IDRs, as well as damaged proteins that have lost their tertiary structures. While the first group, IDR comprising proteins, mainly represents proteins with regulatory and signaling functions that coordinate key cellular processes [13], the second group comprises damaged proteins, which may lead to cytotoxicity and human pathologies and consequently, should be rapidly removed. It is not yet known whether there is any kind of selectivity between these two groups of substrates, and if there is, how is it achieved. Are partially unfolded proteins doomed to death when the 20S proteolytic capacity is enhanced? Several protective mechanisms have been discovered that rescue proteins with IDRs from degradation, such as interactions with “nanny” proteins that mask the unstructured regions [138], or oxidation-mediated structural stabilization [137]; however, the extent to which these processes are widespread is yet to be investigated. Furthermore, it is not yet known how NQO1, which rescues proteins with IDRs from degradation, influences the abundance of damaged, partially unfolded proteins. Similarly, while the presence of damaged proteins triggers 20S proteolytic capacity, it remains to be discovered whether comparable mechanisms exist for regulating the abundance of partially unfolded proteins. Overall, the nature of the molecular cross-talk between 20S proteasome IDPs, and damaged proteins, requires further investigation.

The importance of in-depth exploration of the 20S proteasome degradation pathway, also lies in its potential therapeutic benefits. To date, proteasome inhibitors as bortezomib and carfilzomib have been developed for treating certain cancers, especially multiple myeloma and mantle cell lymphoma [139,140,141], and many other such inhibitors are currently being tested for anti-tumor activities [142]. These drugs, however, target the chymotrypsin-like activity of the 20S proteasome, and inhibit the activities of both the 20S and 26S proteasomes. Thus, it remains to be explored whether selective drug intervention specifically inhibiting either proteasomes will improve the rates of cancer cell toxicity, and/or minimize the deleterious side effects of the current therapeutic regimens. Alternatively, 20S proteasomal activation, which is not only associated with increased lifespan, is also expected to demonstrate clinical utility, considering that oxidatively damaged proteins and protein aggregates are the cause of a large number of neurological diseases [3,8].
